# The non-preventive effects of human menopausal gonadotropins on ovarian tissues in Nandrolone decanoate-treated female rats: A histochemical and ultra-structural study

**Published:** 2018-03

**Authors:** Fakhroddin Mesbah, Hossein Bordbar, Tahereh Talaei Khozani, Farzaneh Dehghani, Hossein Mirkhani

**Affiliations:** 1 *Department of Anatomical Sciences, School of Medicine, Shiraz University of Medical Sciences, Shiraz, Iran.*; 2 *Department of Pharmacology, School of Medicine, Shiraz University of Medical Sciences, Shiraz, Iran.*

**Keywords:** Apoptosis, hMG, Nandrolone decanoate, Ovarian follicle, Rat

## Abstract

**Background:**

The follicular growth and development may be affected by abused drugs. Nandrolone decanoate (ND) as an anabolic androgenic steroid can damage the morphological and functional features of the ovary and may lead to reproductive failure.

**Objective:**

This study was designed to evaluate the effects of synchronized and non-synchronized administration of Human Menopausal Gonadotropins (hMG) with ND on ovarian tissue and level of sex hormones in the adult female rat.

**Materials and Methods:**

Forty adult female Sprague Dawley rats were divided into eight groups. The five experimental groups received 3 and/or 10 mg/kg of ND synchronized and non-synchronized with 10 IU of hMG and hMG alone. The two shams and control groups received solvents of ND and hMG. The animals' serum levels of Follicle-stimulating hormone, Luteinizing hormone, progesterone and estrogen and the weight, volume and dimensions of the ovaries were measured. The ovaries were prepared for apoptosis assessment and morphological study.

**Results:**

The ovarian volume and sex hormones in the experimental groups were decreased, but ovarian weight and dimensions didn’t change. The rate of apoptosis was increased in the experimental groups as follows; a low and high dose of ND synchronized with hMG 48.80±18.70 and 65.20±14.20 respectively vs. Sham 1, 33.20±17.80, a low and high dose of ND non-synchronized with hMD 55.80±17.20 and 75.20±14.30 respectively vs. Sham 2, 31.60±32.40 groups, p˂0.01. Follicular and stromal cells were damaged in the experimental groups except for the hMG group.

**Conclusion:**

Administration of ND decreased the serum level of Luteinizing hormone, Follicle-stimulating hormone, progesterone and estrogen and damaged ovarian tissue irreversibly and irreparably and hMG cannot prevent the destruction of the follicles in the adult female rats. This can be a serious warning to women who abuse ND.

## Introduction

During folliculogenesis the oocyte, granulosa cells (GCs) and theca cells (TCs) remain active in order to attain developmental competence and insure the oocyte survival (1). The growth and development of pre-antral follicles depend on autocrine and paracrine factors but not endocrine factors, such as gonadotropin hormones. However, the growth and development of antral follicles are dependent on gonadotropins (2). The TCs are the source of androgens for GCs to be converted to estrogen (3). The development of antral follicles is affected by follicle-stimulating hormone (FSH) that not only prevents GCs apoptosis and follicular atresia but is also essential for GCs proliferation, production of estradiol and expression of the luteinizing hormone (LH) receptor (4-6). 

Estradiol deficiency can eventually lead to follicular apoptosis and atresia. Androgens are also essential for GCs response to gonadotropins (2). The development of follicles and the maturation of oocytes may be affected by internal factors as well as external factors such as physical, environmental, pharmaceutical, and toxic factors and diseases that lead to many microscopic and ultramicroscopic changes in the follicles. Anabolic androgenic steroid (AASs) and gonadotropins are among the external and internal factors, respectively, affecting folliculogenesis.

The global prevalence of AASs abuse among women is about 1.6% (7). Nandrolone decanoate (ND) is an AAS with great popularity and acceptability among professional and non-professional bodybuilders and athletes. ND is the best-known injectable steroid after testosterone and is considered one of the most effective drugs to maintain lean muscle mass and stimulate the appetite. ND and testosterone are more likely to bind to the androgen receptor in muscle tissue (8). However, it is not recommended for women because of serious side effects such as increased body hair, especially on the face, irregular menstrual cycle, enlargement of the clitoris, deepening of the voice and male pattern alopecia (9, 10). ND results in an increase in the number of atretic follicles in mice and rats, which in turn decreases the number of oocytes (11-13). Through negative feedback, AASs reduce the amount of gonadotropin secretion and thereby affect follicular growth (11). 

As mentioned above, gonadotropin hormones of the pituitary gland are the most important factors affecting folliculogenesis, oocyte maturation, and ovulation. Of these hormones, FSH and LH coordinate the development of antral follicles and ovulation. Human menopausal gonadotropin (hMG) as an alternative for endogenous gonadotropins has an equal amount of FSH and LH and also growth factors. Therefore, it can be a suitable successor for pituitary gonadotropins and is therapeutically used to stimulate folliculogenesis in infertile women and in assisted reproductive technology (14). 

This study was designed to evaluate the synchronized and non-synchronized effects of hMG on ovarian tissue and serum levels of female sex hormones in the ND-treated adult rat.

## Materials and methods

**The animals**

Forty adult female Sprague Dawley rats weighing 180-210 gr were selected from Shiraz University of Medical Sciences (SUMS) animals’ center. The rats were kept in separate cages under standard conditions (12 hr light/dark) and temperature 22±2^o^C and proper moisture. The rats had no experience of pregnancy.

**Study design**

To define sexual cycle phases, the vaginal smear was taken and then the rats that were in estrous phase were randomly divided into eight groups and treated as detailed below:

Group 1: The rats received a low dose of ND (3 mg/kg, volume 0.1 mL) for 4 wk (once a wk) intra-peritoneal, synchronized (Syn) with hMG (10 IU, volume 0.1 mL) for 4 wk (twice a wk) intra-muscular (Low dose nandrolone decanoate (LDND)+hMG Syn).

Group 2: The rats received a high dose of ND (10 mg/kg, volume 0.1 mL) for 4 wk (once a wk) intra-peritoneal, Syn with hMG (10 IU, volume 0.1 mL) for 4 wk (twice a wk) intra-muscular (High dose nandrolone decanoate (HDND)+hMG Syn).

Group 3: The rats received olive oil (solvent of ND), 0.1 mL for 4 wk (once a wk) intra-peritoneal, Syn with sodium chloride 0.9% (solvent of hMG) 0.1 mL for 4 wk (twice a wk) intra-muscular (Sham 1).

Group 4: The rats received LDND (3 mg/kg, volume 0.1 mL) for 4 wk (once a wk) intra-peritoneal, Non-Syn with hMG (10 IU, volume 0.1 mL) two wk after the last injection of ND for 2 wk (twice a wk) intra-muscular (LDND+hMG Non-Syn).

Group 5: The rats received HDND (10 mg/kg, volume 0.1 mL) for 4 wk (once a wk) intra-peritoneal, Non-Syn with hMG (10 IU, volume 0.1 mL) two wk after the last injection of ND for 2 wk (twice a wk) intra-muscular (HDND+hMG Non-Syn). 

Group 6: The rats received olive oil, 0.1 mL for 4 wk (once a wk) intra-peritoneal, and sodium chloride 0.9%, 0.1 mL two wk after the last injection of olive oil for 2 wk (twice a wk) intra-muscular (Sham 2).

Group 7: The rats received hMG (10 IU, volume 0.1 mL) for 4 wk (twice a wk) intra-muscular (hMG). Because of the shorter half-life of hMG than the ND, in order to obtain sufficient effect of hMG, it was used twice weekly in experimental groups.

Group 8: The rats received sodium chloride 0.9%, 0.1 mL for 4 wk (twice a wk) intra-muscular (Control).

**Ovarian weight, volume, length and width**

After sacrifice of each animal, the ovaries were removed and cleaned and then weight, volume (based on the Archimedes' principle), length and width were measured to an accuracy of 0.01. To measure the ovarian weight and volume, the digital scale (Scaltec Spo51 Germany) and for ovarian dimensions, the digital caliper manual (Mitutoyo) were used.

**Hormonal assay**

The serum levels of FSH, LH (Shibayagi Tokyo Japan), estrogen and progesterone (CUSABIO China) were assessed by rat ELISA Kit, two times, the first time was before doing any treatment and the second time before sacrificing the animals. The blood samples were collected from the rat's tails and centrifuged at 4^o^C for 10min at 250g. The serums were kept at -20^o^C until the biochemical analysis. The samples were incubated in monoclonal anti-LH/FSH/ estrogen/progesterone antibody and measured spectrophotometrically at 450 nm (15). 

**Histological preparation**

The right ovary of each animal was put in 10% formalin for 24 hr in automatic tissue processor and then blocked by paraffin with the regular method. The block was cut into sections of 5 µm thickness (MICRON HM 325 Germany) and stained with hematoxylin and eosin and observed by light microscopy.

**TUNEL method**

The TUNEL method was used for the reaction of histochemistry and the detection of apoptosis. The left ovaries were immediately immersed in 4% paraformaldehyde for 24 hr. Paraffin blocks were prepared using regular tissue processing method, then the 5µm sections were deparaffinized at 37-40^o^C temperature. The sections were rehydrated in descending series of ethanol, and treated with 3% hydrogen peroxide in methanol at room temperature for 5 min, after each step they were washed in phosphate buffer saline (PBS). Tissue sections were incubated with proteinase K (Sigma, Germany) 3 µl/mL in Tris- HCl buffer (Merck, Germany) in humidity condition at 37^o^C for 30 min and rinsed twice in PBS for 10 min. The DNA fragments were localized using peroxidase apoptotic detection kit (POD Kit, Roche, Germany) based on the manufacturer’s protocol. 

The sections were treated with antibody conjugated with fluorescein, in dark and humid conditions. The sections were assessed with fluorescent microscopy (Nikon, E 600, Japan) at a wavelength of 450-490 A. The bright green spots represent apoptotic cells. For light microscopic detection of cellular apoptosis, the sections were stained with diaminobenzidine solution (20 µL) then dehydrated and mounted (16).

**Determining the percentage of apoptosis**

After TUNEL staining 10 slides were selected from each group and observed by a light microscope with 20× magnification, and the actual number of brown nuclei of oocytes (signs of apoptosis) in the primordial follicles were counted. The healthy follicles were counted directly as well. The apoptotic index was calculated by the following equation:

“Apoptosis percentage= the number of apoptotic primordial follicles÷total number of primordial follicles×100”.

**Tissue preparation for TEM**

The left ovaries were trimmed and placed in 2.5% glutaraldehyde for 2-3 hr and then segmented into pieces of 1mm^3^. The tissue segments were primary-fixed in 2.5% glutaraldehyde overnight and post-fixed in 1% buffered osmium tetroxide for 1 hr, after each fixation the samples were washed three times with PBS. This was followed by dehydration of the segments in graded series of 30-100% ethanol and infiltration by different ratios; 1 to 3, 1 to 1 and 3 to 1 of resin/ethanol and pure resin and embedded by resin (Agar 100). The blocks were polymerized overnight at 60^o^C in the oven. The semi-thin sections (1μm) were prepared and stained with 1% toluidine blue and previewed by light microscope. The ultra-thin sections (60-90 nm) were prepared, and stained with uranyl acetate and lead citrate and observed by TEM (Philips CM 10) (17). All chemicals were obtained from Sigma.

**Ethical consideration**

The study was approved by the SUMS ethics committee guidelines (2017-351).

**Statistical analysis**

Data were expressed as the mean±S.D. The data were analyzed through the Kruskal-Wallis non-parametric test. A p<0.01 was considered statistically significant. The statistical analyses were done using the SPSS statistical software, version 15.0 (Statistical Package for the Social Sciences, SPSS Inc, Chicago, Illinois, USA).

## Results

**Ovarian weight, volume, length and width**

The mean weight of the ovaries in the experimental groups compared with the sham and control groups did not differ significantly. The mean length and width of the ovary did not differ significantly between the groups. Ovarian volume decreased significantly in the HDND+hMG (Syn) group compared with Sham 1, in the LDND and HDND+hMG (Non-Syn) groups compared with Sham 2 and in the hMG group compared with the control group (p<0.01, Table I).

**Hormones**

The serum levels of FSH, LH, estrogen, and progesterone decreased significantly in both LDND and HDND+hMG (syn) groups compared with Sham 1 (p˂0.01). The serum levels of FSH, LH, estrogen and progesterone decreased significantly in both LDND and HDND+hMG (non-syn) groups compared with Sham 2, except estrogen in the LDND+hMG (non-syn) group (p˂0.01). Estrogen levels increased significantly in the hMG group compared with the control group (p˂0.01), Table II).

**Histology of the ovary**

**Figure 1:**
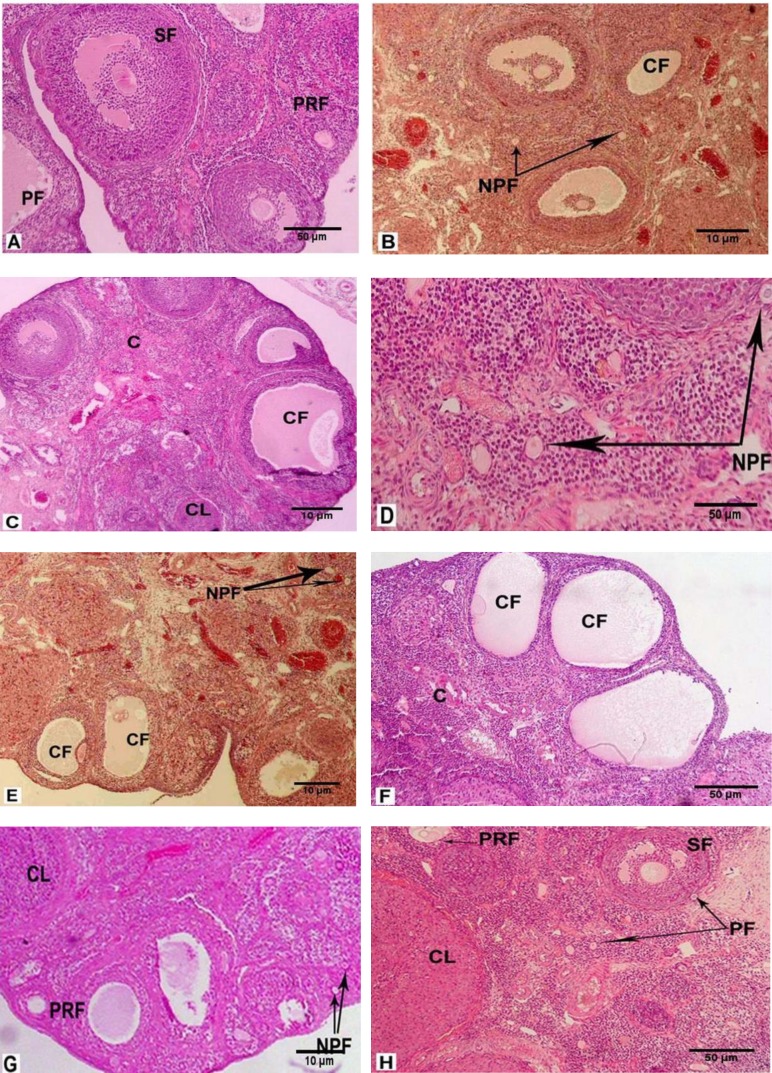
Histology of the ovary in the rats that received: no treatment (A, 100×), LDND synchronized with hMG (B, 40×), HDND synchronized with hMG (C, 40×), solvents of ND and hMG synchronized (D, 100×), LDND non-synchronized with hMG (E, 40×), HDND non-synchronized with hMG (F, 100×), solvents of ND and hMG non-synchronized (G, 40×), hMG (H, 100×). Hematoxylin-eosin staining.

The types of all follicles and corpus luteum (CL) were normal in the control group and no cystic and atretic follicles were found (Figure 1-A). In both the LDND and HDND+hMG (syn) groups (Figure 1-B, C), lower hyperemia CL was observed than in the Sham 1 group (Figure 1-D). Moreover, cystic follicles were observed in the HDND+hMG (syn) group (Figure 1-C). More hyperemia CLs were observed in the LDND and HDND (Figure 1-E, F) +hMG (non-syn) groups than in the Sham 2 group (Figure 1-G). The cystic follicles, particularly in the HDND+hMG (non-syn) were observed (Figure 1-F). In the hMG group (Figure 1-H), compared with the control group, no significant changes were observed and some types of normal follicles with CL and a few atretic and cystic follicles were seen. 

**Histochemistry of ovarian tissue**

**Figure 2:**
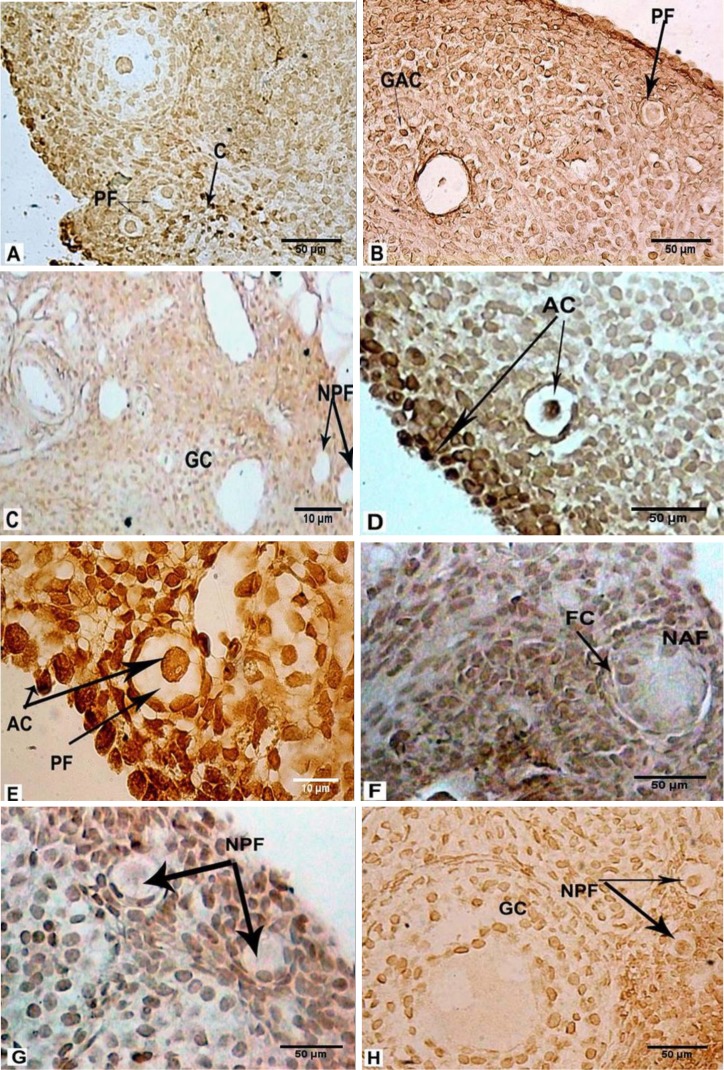
Histochemical evaluation of the ovary in the rats that received: LDND synchronized with hMG (A, 100×), HDND synchronized with hMG (B, 100×), solvents of ND and hMG synchronized (C, 40×), LDND non-synchronized with hMG (D, 100×), HDND non-synchronized with hMG (E, 400×), solvents of ND and hMG non-synchronized (F, 100×), hMG (G, 100×), no treatment (H, 100×). The sections were stained with diaminobenzidine.

**Figure 3:**
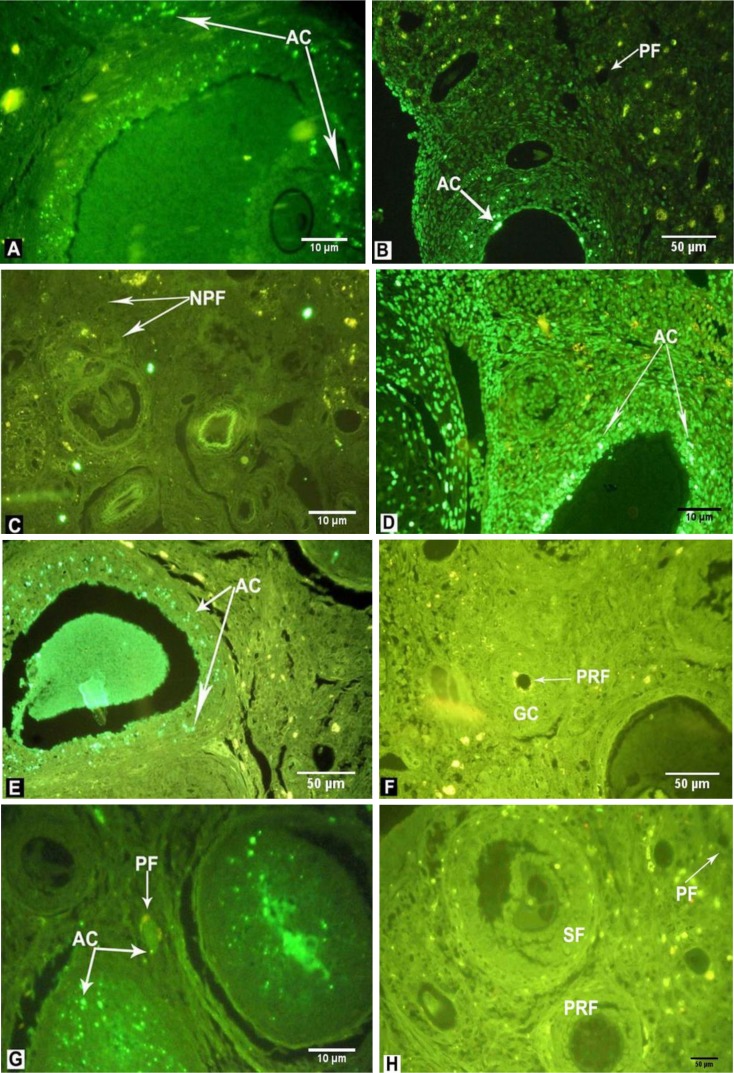
Fluorescent microscopic evaluation of the ovary in the rats that received: LDND synchronized with hMG (A, 400×), HDND synchronized with hMG (B, 100×), solvents of ND and hMG synchronized (C, 40×), LDND non-synchronized with hMG (D, 400×), HDND non-synchronized with hMG (E, 100×), solvents of ND and hMG non-synchronized (F, 100×), hMG (G, 400×), no treatment (H, 100×). The sections were treated with antibody conjugated with fluorescein.

The percentage of apoptosis in primordial follicles was calculated as detailed below and in table III. Comparison of apoptosis in both the LDND (Figures 2-A and 3-A) and HDND (Figures 2-B and 3-B) +hMG (syn) groups with the Sham 1 (Figures 2-C and 3-C) showed a significant increase (p˂0.01). The rate of apoptosis increased significantly in both the LDND (Figures 2-D and 3-D) and HDND (Figures 2-E and 3-E) +hMG (non-syn) groups compared with the Sham 2 (Figures 2-F and 3-F) and hMG groups (p˂0.01). Also, apoptosis was observed in GCs in these two groups. The percentage of apoptosis in the hMG (Figures 2-G and 3-G) group compared with other groups, including control (Figures 2-H and 3-H) showed a significant reduction (p˂0.01); GCs apoptosis was observed less in this group.

**Histological and ultra-structural features of follicles in the sham and control groups**

**a. Histology**

**Figure 4:**
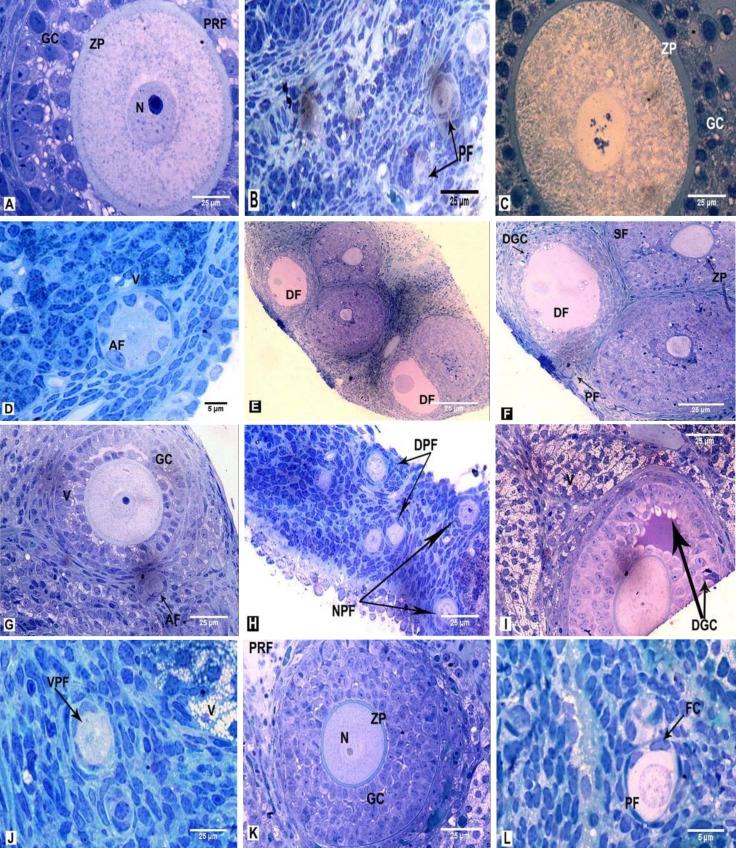
Histology of the resin-embedded ovary in the rats that received: no treatment and solvents of ND and hMG synchronized (A, B, 400×both), LDND synchronized with hMG (C, 400×, D, 600×), HDND synchronized with hMG (E, F, 200× both), LDND non-synchronized with hMG (G, H, 400× both), HDND non-synchronized with hMG (I, J, 400× both), hMG (K, 400×, L, 100×). Toluidine blue staining.

Pre-antral follicles with healthy GCs and their dark and light nuclei, normal TCs, oocyte nucleus, and nucleoli were identified and accompanied by constant zona pellucida (ZP). The numbers of healthy primordial follicles were noticed (Figures 4-A, B).

**b. Ultra-structure**

**Figure 5:**
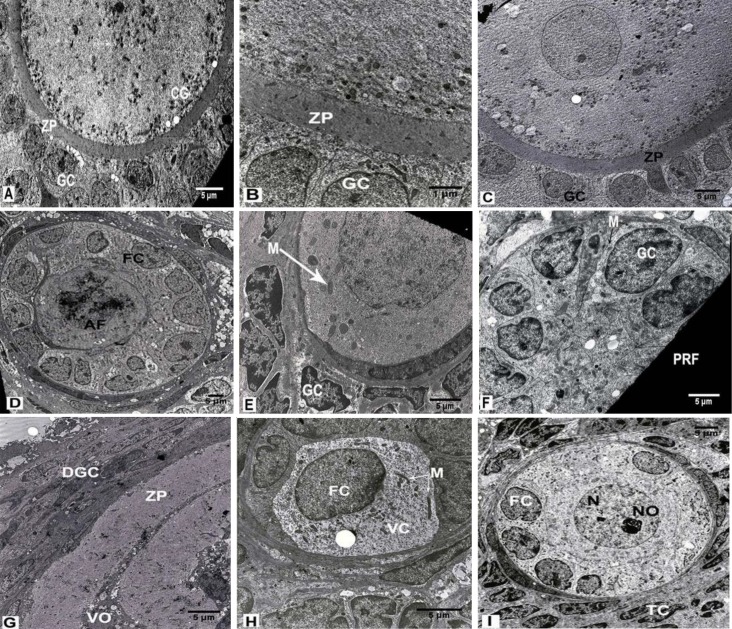
Ultra-structural features of the ovary in the rats that received: no treatment and solvents of ND and hMG synchronized (A, 1200×, B, 3900×), LDND synchronized with hMG (C, 2200×), HDND synchronized with hMG (D, 1200×), LDND non-synchronized with hMG (E, F, 2950× both), HDND non-synchronized with hMG (G, H, 2200× both), hMG (I, 1650×). Uranyl acetate and lead citrate staining.

Normal GCs with light and dark rounded and elongated nuclei, as well as the defined cytoplasmic membrane and cells junctions, were observed. The GCs contained normal vesicular and tubular rough endoplasmic reticulum (RER) without any dilation, round and elongate shaped mitochondria (Mt) with distinct crista as well as Golgi complexes and normal configuration of chromatin. There were no vacuoles in GCs and TCs. In some instances, primordial and primary follicles were observed. Even and clear ZP was recognized in primary follicles. Narrow perivitelline space contained thin microvilli and cumulus cells process endings. In the ooplasm, Mt was regularly distributed and structures like cortical granule were noticed (Figures 5-A, B).

**Histological and ultra-structural features of follicles in LDND+hCG (syn) and HDND+hCG (syn) groups**

**a. Histology**

In the group that received a low dose of ND, a number of follicles and healthy GCs were observed. Ooplasm was homogenous and was vacuolated in some cases and normal ZP thickness was observed. Vacuoles were also observed in ovarian stroma. In general, the number of primordial follicles and other types of follicles were more than the Sham 1 and control groups (Figures 4-C, D). In the group that received a high dose of ND, a number of follicles with destroyed GCs were seen. In some follicles, unchanging ZP with ooplasm was seen. There were different sizes and shapes of GCs in the follicles. The healthy primordial activated and other types of follicles with CL were also observed (Figures 4-E, F).

**b. Ultra-structure**

In the group that received a low dose of ND, little vacuoles were seen in GCs and TCs. The chromatin condensation of GCs was less, the nuclei were different with respect to shape and size, and the cell membrane and its junctions were observed. Activated follicles with reticular nucleus were observed. Homogenous ooplasm with a normal distribution of the organelles was noticed (Figure 5-C).

In the group that received a high dose of ND, many vacuoles were seen in GCs and TCs, and their nuclei had different shapes and sizes and less compact chromatin was defined. The cell membrane and junctions were visible, a number of Mt in different shapes and sizes with defined crista and vesicular RER were visible. In some samples, the primordial and primary follicles were founded. In some of the primary follicles, the nucleus of GCs was pyknotic. The ZP was uniform and perivitelline space was not visible. In some cases, the ooplasm contained vesicular and somewhat distended RER and Mt with distinct crista were clearly seen (Figure 5-D). 

**Histological and ultra-structural features of follicles in LDND+hCG (non-syn) and HDND+hCG (non-syn) groups**

**a. Histology**

In the group that received a low dose of ND, light and dark GCs and vacuolated ooplasm were seen. Numbers of different categories of healthy follicles and normal ZP were observed. Also, the activated follicles with a reticular nucleus and abundant vacuoles were observed in ovarian stroma (Figures 4-G, H). In the group that received a high dose of ND, there were healthy and damaged vacuolated follicles. Some GCs were destroyed and vacuolated and the ovarian stroma contained many vacuoles (Figures 4-I, J).

**b. Ultra-structure**

In the group that received a low dose of ND, there were more vacuoles in GCs and TCs than the Sham 2 group. The primary follicles, along with GCs with elongated nuclei and constant chromatin were observed. In the ooplasm, the numbers of Mt in different shapes and sizes and defined crista and all the cytoplasmic organelles were seen. The ZP was not formed and the membrane of GCs and the junctions between them were obvious (Figures 5-E, F).

In the group that received a high dose of ND, abundant vacuoles in various sizes were seen in GCs and TCs. The nuclei of GCs were in different shapes and irregular and uncertain boundaries of the cells were noticed and most of them were damaged. A number of abnormal curved Mt with irregular crista, vesicular smooth endoplasmic reticulum and lipid droplet were detected in GCs. In some cases, follicles were destroyed along with non-uniform ZP and ooplasmic vacuolization was found (Figures 5-G, H).

**Histological and ultra-structural features of follicles in hMG group**

**a. Histology**

In this group, the healthy secondary follicles were observed with GCs and TCs. The constant ZP in a variety of healthy follicles, as well as a number of healthy primordial follicles, were observed (Figures 4-K, L).

**b. Ultra-structure**

A number of healthy activated follicles were observed. The GCs with different forms of the vesicular endoplasmic reticulum, normal cell membranes and their junctions were seen. Ooplasm components were normal and ZP had not formed. Resting oocyte nucleus and nucleoli were clearly seen. In some instances, healthy primordial follicles with normal oocyte and monotonous chromatin were seen. TCs in secondary follicles were normal (Figure 5-I).

**Table I:** Ovarian weight, dimensions and volume

**Groups**	**Ovary weight (mg)**	**Ovary length (mm)**	**Ovary width (mm)**	**Ovary **v**olume (mm**^3^**)**
LDND + hMG (Syn)	30.00 ± 15.00	4.50 ± 1.30	2.80 ± 1.00	20 ± 10.00
HDND + hMG (Syn)	30.00 ± 10.00	4.90 ± 0.60	2.70 ± 1.00	16 ± 5.48^a^
Sham 1 (Syn)	30.00 ± 13.00	4.50 ± 0.60	3.30 ± 0.40	20 ± 10.00
LDND + hMG (Non-Syn)	30.00 ± 8.00	5.30 ± 0.60	3.60 ± 0.30	24 ± 5.48^b^
HDND + hMG (Non-Syn))	30.00 ± 13.00	4.50 ± 1.30	3.50 ± 0.50	24 ± 8.94^b^
Sham 2 (Non-Syn))	30.00 ± 7.00	5.00 ± 0.40	3.30 ± 0.40	28 ± 8.37
hMG	30.00 ± 8.00	4.40 ± 0.40	2.30 ± 0.40	24 ± 8.94^c^
Control	30.00 ± 4.00	5.01 ± 0.30	3.410± 0.30	28 ± 8.47

Data presented as mean±SD.

LDND: Low dose nandrolone decanoate

hMG: Human menopausal gonadotropins

HDND: High dose nandrolone decanoate

a Decreased significantly compared with the sham 1 (p<0.01).

b Decreased significantly compared with the sham 2 (p<0.01).

c Decreased significantly compared with the control (p<0.01).

**Table II:** Serum levels of FSH, LH, estrogen and progesterone

**Groups**	**FSH (ng/mL)**	**LH (ng/mL)**	**Estrogen (ng/mL)**	**Progesterone (ng/mL)**
LDND + hMG (Syn)	1.98 ± 0.42 ^a^	6.70 ± 0.44 ^a^	25.5 ± 1.16 ^a^	7.20 ± 0.38^ a^
HDND + hMG (Syn)	2.16 ± 0.64 ^a^	6.32 ± 0.41^a^	25.7 ± 1.10 ^a^	7.10 ± 1.01^ a^
Sham 1 (Syn)	2.99 ± 0.45	7.72 ± 0.45	27.01 ± 0.86	8.10 ± 0.44
LDND + hMG (Non-Syn)	2.23 ± 0.60 ^b^	6.96 ± 0.58 ^b^	26.02 ± 0.66	7.30 ± 0.26 ^b^
HDND + hMG (Non-Syn)	1.47 ± 0.39 ^b^	6.80 ± 0.81 ^b^	24.70 ± 1.50 ^b^	7.10 ± 1.06 ^b^
Sham 2 (Non-Syn))	3.11 ± 0.36	8.01 ± 0.25	26.50 ± 1.21	8.00 ± 0.19
hMG	3.24 ± 0.37	8.08 ± 0.27	27.80 ± 1.00^c^	8.20 ± 0.51
Control	3.01 ± 0.29	7.81 ± 0.50	26.10 ± 0.47	7.80 ± 0.53

Data presented as mean±SD.

LDND: Low dose nandrolone decanoate

hMG: Human menopausal gonadotropins

HDND: High dose nandrolone decanoate

FSH: Follicle-stimulating hormone

LH: Luteinizing hormone

a Decreased significantly compared with the Sham 1 (p<0.01).

b Decreased significantly compared with the Sham 2 (p<0.01).

c Increased significantly compared with the control (p<0.01).

**Table III:** Percentage of apoptosis in experimental, sham and control groups

**Groups**	**Apoptosis (%)**
LDND + hMG (Syn)	48.80 ± 18.70^a,^^f^
HDND + hMG (Syn)	65.20 ± 14.20^a, ^^f^
Sham 1 (Syn)	33.20 ± 17.80
LDND + hMG (Non-Syn)	55.80 ± 17.20^b^^,^^c^^, ^^f^
HDND + hMG (Non-Syn)	75.20 ± 14.30 ^b^^,^^d^^, ^^f^
Sham 2 (Non-Syn)	31.60 ± 32.40
hMG	29.80 ± 21.80^e^
Control	32.20 ± 16.90

Data presented as mean±SD.

an Increased significantly compared with the Sham 1 (p<0.01).

LDND: Low dose nandrolone decanoate

hMG: Human menopausal gonadotropins

HDND: High dose nandrolone decanoate

b Increased significantly compared with the Sham 2 (p<0.01).

c Increased significantly compared with the LDN+hMG (Syn) (p<0.01).

d Increased significantly compared with the HDN+hMG (Syn) (p<0.01).

e Decreased significantly compared with the control (p<0.01).

f Increased significantly compared with the sham groups and control (p<0.01).

SF: Secondary follicle

PRF: Primary follicle

PF: Primordial follicle

NPF: Normal primordial follicle

CF: Cystic follicle

C: Congestion

CL: Corpus luteum.

LDND: Low dose nandrolone decanoate

HDND: High dose nandrolone decanoate

AC: Apoptotic cell

PF: Primordial follicle

GAC: Granulosa apoptotic cell

NPF: Normal primordial follicle

GC: Granulosa cell

AC: Atretic cell

FC: Follicular cell

NAF: Normal activated follicle

LDND: Low dose nandrolone decanoate

HDND: High dose nandrolone decanoate

AC: Atretic cell

PF: Primordial follicle

NPF: Normal primordial follicle

PRF: Primary follicle

SF: Secondary follicle

LDND: Low dose nandrolone decanoate

HDND: High dose nandrolone decanoate

AF: Activated follicle

PF: Primordial follicle

NPF: Normal primordial follicle

PRF: Primary follicle

SF: Secondary follicle

GC: Granulosa cell

ZP: Zona pllucida

N: Nucleus

V: Vacuole

DF: Degenerated follicle

DGC: Degenerated granulosa cell

VPF: Vacuolated primordial follicle

FC: Follicular cell.

LDND: Low dose nandrolone decanoate

HDND: High dose nandrolone decanoate

PRF: Primary follicle

GC: Granulosa Cell

ZP: Zona Pllucida

N: Nucleus

V: Vacuole

DGC: Degenerated granulosa cell

FC: Follicular cell

CG: Cortical granule

AF: Activated follicle

M: Mitochondria

VO: Vacuolated ooplasm

VC: Vacuolated cytoplasm

NO: Nucleolus

LDND: Low dose nandrolone decanoate

HDND: High dose nandrolone decanoate

## Discussion

The present study showed that the AASs such as ND effect on the structure and function of the reproductive system. AASs increase the appearance and functions of GABA-A receptors in the diencephalon and could lead to a change in sexual behavior and reproductive biology (18). GABA-A receptors control the HPG axis by regulating the tonic secretion of gonadotropin-releasing hormone. Therefore, AASs may alter the activity of cells in this area and may also result in the release of VEGF. While both estrogen receptor and the androgen receptor signalling pathway are clearly involved as mediators in AASs performance, GABA circuits in the diencephalon are key regulators of HPG axis. The GABA receptor function is affected by hormone receptors, hormone metabolism, and the levels of steroids in the body (18).

The increased prevalence of AASs use and its irreparable side effects among the abusers, lead to self-administration of anti-androgen drugs, in order to prevent and eliminate these damages (19).

The results of this study suggest that the synchronized and non-synchronized administration of hMG with ND or alone has no effects on ovarian weight and dimensions of the experimental groups compared with the control and sham groups. In contrast, the findings of Simao *et al* have shown the reduction in ovarian weight following administration of a different dose of ND treatment (11), also these results are confirmed by Camargo *et al* findings (20). Some studies have demonstrated that ND has a negative feedback on the HPG axis, which leads to decreased levels of FSH, LH, progesterone and estrogen hormones and the number of follicles, and consequently reduces the volume of the ovary (11, 13), as it is shown in this study and in a previous study (21). Reduction in the ovarian volume is caused by a decrease in the number of follicles, so the evaluation of ovarian volume is a key point for understanding the functions of the ovaries (22). 

Our study showed that synchronized co-administration of hMG with low-dose of ND in comparison with high-dose of ND and sham, prevents a decrease in ovarian volume, while non-synchronized administration of hMG is not sufficient to prevent the reduction in ovarian volume. These results reflect the irreversible destruction of ovarian tissue after taking ND. Simao *et al* concluded that high dose of ND (15 mg/kg BW) damaged rat ovarian and uterine tissue and decreased the rate of fertility to the extent, that they were not restored after a recovery period of 30 to 60 days (11). Therefore, it can be said that the female reproductive system is more vulnerable to AASs, compared with males. Because it is demonstrated that testicular atrophy is reversible after the discontinuation of ND (23). 

Increased ovarian volume occurs when the antral follicles are formed, which is dependent on the gonadotropin hormones. In our study, since the synchronized and non- synchronized administration of hMG with ND could not compensate FSH and LH deficiency that emerged with the use of ND, the reduction of the ovarian volume in the experimental groups was seen, except in the LDND+hMG (Syn) group, especially in the groups that received non-synchronized hMG with both low and high doses of ND. These results could be attributed to the insufficient amount of hMG, less hMG half-life, duration of treatment, high durability and long half-life of ND in the body, more destructive effects of ND, and presence of serious destruction in ovarian follicles. Bagchus *et al* have reported that metabolites of ND were detectable up to six months after the injection of a high dose of ND (24). Most likely, in the rats that received a high dose of ND, more amounts of the hMG and/or co-administrated with other substances are required in order to avoid the adverse effects of ND. In the clinic, hMG is administrated to stimulate ovary and prevent reduction of ovarian volume, especially co-administrated with clomiphene citrate (25), and has positive effects on oocyte fertilization (26).

In this study, ND reduces the serum levels of FSH, LH, estrogen and progesterone in the experimental groups, and synchronized and non-synchronized administration of hMG with ND had no effect on it, while the administration of hMG alone somewhat increased the level of FSH, LH, progesterone and estrogen. According to the results of Shirwalker *et al* the administration of estradiol decreases gonadotropins in female rats (27). It seems that the effects of ND on the HPG axis cannot be compensated by hMG. Moreover, this hMG disability can be the reason for the low level of FSH in HDND+hMG (Non-syn) group in comparison with HDND+hMG (syn) group, furthermore, it may be due to the non-synchronized administration of hMG with ND as well. Reduction of the gonadotropins in the experimental groups and inability of hMG to compensate these hormones leads to decreased estrogen and progesterone levels (28). 

Most likely, the anti-estrogenic and anti-progesterone effects of ND, through reducing the expression and activity of receptors on the ovary, contribute to the decreased levels of estrogen and progesterone. Also, the irreversible damage of TCs in the ovary facilitates this process. The reduction in these hormones and ineffectiveness of exogenous gonadotropins in repairing ovarian tissue puts women who consume steroidal compounds at high risk. In our study, lack of adequate treatment, an impure growth factor of hMG (29), low dose of hMG and the short duration of treatment were among the reasons sex hormones did not increase after the administration of hMG. This is in agreement with Tabata *et al* who demonstrated that hMG cannot increase the recovery rate of oocyte and serum level of gonadotropins in human (30). In humans, combinations of pharmacological protocols are often used to stimulate the ovary. Wolff *et al* reported that some growth factors which are contained in hMG, such as epidermal growth factor, may interfere with other growth factors that play an essential role in the follicular development and suppress estrogen production (29). 

In our study, follicular apoptosis in experimental groups, especially in the rats that received a high dose of ND, had increased so much that synchronized and non-synchronized administration of hMG with ND have failed to prevent it. These results are similar to those of Simao’s study that demonstrated that the ND induces follicular atresia, which is a dose-dependent effect and may influence neuroendocrine axis and represent androgen receptor on GCs and TCs (11). These effects cause follicular apoptosis and ovarian degeneration and atrophy and consequently reduce the volume of the ovary, as we observed in the both this and previous stereological studies (21). The studies indicate that follicular atresia occurs after AASs abuse (11, 12). ND affects the HPG axis and reduces the influence of endocrine factors affecting follicular maturation such as FSH and the resulting LH. The presence of atresia could decrease autocrine and paracrine factors that affect folliculogenesis, resulting in increased numbers of atretic antral and pre-antral follicles. In this study, hyperemia was observed in the groups that received synchronized hMG with ND, which represents the positive effect of hMG, as Becker *et al* have shown that steroids have an anti-angiogenic role (31). 

Wang *et al* showed that hMG increases blood flow to the follicles and their growth factors in mice through increased expression of vascular endothelium growth factor (32). Also, AASs (testosterone) aromatized into estradiol which results in dilation of vessels (11). Increase in the rate of apoptosis in the groups that received synchronized hMG with ND showed that apoptosis has a dose-dependent manner with respect to ND, and the hMG dosage we used was ineffective in preventing atresia. Decreased serum level of sex hormones, especially FSH and LH can increase the rate of apoptosis. Most likely, to preserve the stability of gonadotropin levels in the blood of AASs abusers in the groups that received non-synchronized administration of hMG with ND, the further dosage of hMG and/or purified hMG is required (29). 

Based on the ultra-structural results of this study, apoptosis was approved in the primordial follicles and ovarian stromal cells. It can be concluded that the toxic effects of AASs have an impact on the reduction of follicular growth factors. Also, there are many factors involved in the incidence of apoptosis in primordial follicles and reduction of their numbers. These results are similar to several other studies on mice and rats (33, 34). The previous study reported that intensive follicular atresia tends to differentiate the GCs into Sertoli-like cells (11). Also administration of a combination of ND, testosterone and nicotine results high circulating level of androgens which leads to inducing the ovarian Sertoli cell-like tumors (20) The ultra-structural changes in the rats that received high dose of ND were such that the hMG was not able to fully compensate the damage induced by ND, especially in those that received non- synchronized hMG. However, hMG alone well-maintained the normal ultra-structures of follicles compared with the control and sham groups. 

The morphological and hormonal outcomes of this paper show that high dose of ND caused ovarian tissue damage and reduction of sex hormones in adult female rats. Also, the administration of 10 IU of hMG, especially in those groups that received non- synchronized hMG, cannot prevent these changes. These results are consistent with our previous findings in a stereological and hormonal assay study that demonstrated ND reduced the volume of ovaries, a number of follicles and sex hormone in female rats and hMG could prevent these reductions in those rats that received a low dose of ND (21). It seems that administration of more doses of hMG and co-administration with other substances, such as clomiphene citrate or pure FSH tend to be more beneficial.

## Note

***This article extracted form Ph.D. thesis. (Hossein Bordbar)***
